# Is EC class predictable from reaction mechanism?

**DOI:** 10.1186/1471-2105-13-60

**Published:** 2012-04-24

**Authors:** Neetika Nath, John BO Mitchell

**Affiliations:** 1 Biomedical Sciences Research Complex and EaStCHEM School of Chemistry, Purdie Building, University of St Andrews, North Haugh, St Andrews, Scotland KY16 9ST, UK

## Abstract

**Background:**

We investigate the relationships between the EC (Enzyme Commission) class, the associated chemical reaction, and the reaction mechanism by building predictive models using Support Vector Machine (SVM), Random Forest (RF) and k-Nearest Neighbours (kNN). We consider two ways of encoding the reaction mechanism in descriptors, and also three approaches that encode only the overall chemical reaction. Both cross-validation and also an external test set are used.

**Results:**

The three descriptor sets encoding overall chemical transformation perform better than the two descriptions of mechanism. SVM and RF models perform comparably well; kNN is less successful. Oxidoreductases and hydrolases are relatively well predicted by all types of descriptor; isomerases are well predicted by overall reaction descriptors but not by mechanistic ones.

**Conclusions:**

Our results suggest that pairs of similar enzyme reactions tend to proceed by different mechanisms. Oxidoreductases, hydrolases, and to some extent isomerases and ligases, have clear chemical signatures, making them easier to predict than transferases and lyases. We find evidence that isomerases as a class are notably mechanistically diverse and that their one shared property, of substrate and product being isomers, can arise in various unrelated ways.

The performance of the different machine learning algorithms is in line with many cheminformatics applications, with SVM and RF being roughly equally effective. kNN is less successful, given the role that non-local information plays in successful classification. We note also that, despite a lack of clarity in the literature, EC number prediction is not a single problem; the challenge of predicting protein function from available sequence data is quite different from assigning an EC classification from a cheminformatics representation of a reaction.

## Background

### Encoding enzyme reactions and mechanisms

Almost all biological processes proceed at a significant rate only because of enzymes, proteins that catalyse the chemical reactions found in nature. For half a century, enzymes have been annotated using Enzyme Commission (EC) numbers [1]. The scheme is a hierarchical organization of enzyme reactions into six main classes (oxidoreductases, transferases, hydrolases, lyases, isomerases and ligases), which are then split at a further three hierarchical levels. In general, these successive levels describe the reaction at increasingly fine levels of granularity. The six top level classes are very broad reaction types. The second level subclass and third level sub-subclass usually describe the specific bonds or functional groups involved in the reaction. The fourth level serial number defines the actual substrate and therefore the specific chemical reaction catalysed. The EC classification can be conveniently browsed and searched *via *the ExplorEnz database [2,3], while the official website maintained by the Nomenclature Committee of the International Union of Biochemistry and Molecular Biology (NC-IUBMB) [4] is a valuable and regularly updated resource. Numerous other online databases allow the user to explore enzyme structure and function, including the Enzyme Structures Database [5], IntEnz [6], BRENDA [7] and KEGG [8,9].

Our motivation is to investigate the relationship between the reaction mechanism as described in the MACiE [10-13] (Mechanism, Annotation and Classification in Enzymes) database and the main top-level class of the EC classification. In order to do this, we generate supervised machine learning models to predict EC class from data on the chemical reaction or its mechanism. We consider two ways of encoding the mechanistic information in descriptors, and also three approaches that encode only the overall chemical reaction. These five sets of descriptors are described in detail in the Methods section, and also in Additional file 1, Additional file 2, Additional file 3, Additional file 4, Additional file 5, Additional file 6.

By definition, the EC number describes the overall chemical transformation. Thus a full description of the overall chemical transformation from starting materials to products should, in principle, lead to perfectly accurate assignment of the EC number. However, our objective is not to create perfect cheminformatics algorithms to assign EC numbers, but rather to look at descriptor definitions that encode mechanism-rich information. If similar reactions have a strong tendency to proceed by similar mechanisms, then we would expect descriptors of the mechanism to be (almost) as good as descriptors of the transformation in predicting or assigning EC class. If, however, similar reactions proceed by diverse mechanisms, then we would expect mechanistic descriptors to be relatively poor predictors of EC class.

Work by O'Boyle *et al. *[14] showed that multi-step enzyme catalysed reaction mechanisms can be represented by descriptors, and that a procedure analogous to bioinformatics sequence alignment can be used to measure similarity between stepwise reaction mechanisms. Their study presented instances where quite different chemical transformations were carried out by similar mechanisms. Almonacid *et al. *[15] built on that work with a study of the mechanisms of analogous enzymes, that is pairs of enzymes which catalyse similar overall reactions (identical up to the third EC level) while sharing no detectable common evolutionary ancestry. They showed that the mechanisms catalysed by analogous enzymes are less similar than their overall chemical transformations - demonstrating that convergent evolution typically uses a different mechanism when reinventing an existing kind of enzyme chemistry. This suggests that there are plural chemical mechanisms available to effect a given variety (EC sub-subclass) of overall transformation. This is true despite the usual constraints of approximately pH neutral aqueous chemistry catalysed by a limited alphabet of amino acids and cofactors. In this context we note that, though 14 of the 320 mechanisms in MACiE 3.0 were taken from extremophiles, all but two (M0225 and, debatably, M0123) of these have close homologues in mesophiles that are highly likely to share the same mechanism. These two studies [14,15] motivate our interest in investigating the extent to which predicting EC number from *mechanism *is possible.

### A brief history of EC prediction

Numerous papers have attempted EC prediction, but on the basis of quite different information and with different scientific objectives. It is helpful to divide them into those predictions based on protein properties, and those based on the chemical changes taking place in the reactions. A number of groups have attempted to use *protein sequence and structure *to predict EC number [16-20]. A different class of computational methods tries to automatically link *overall chemical reactions *to EC numbers, not considering mechanism and ignoring protein sequence and structure [21-25].

Firstly, we take the bioinformatics problem of protein function prediction, where predicting an EC number from a previously unseen protein sequence or structure is the objective. Bray, Doig & Warwicker [16] constructed a predictive model based on a combination of sequence, structural and active site features of a diverse set of enzymes. Their model operates by generating a vector describing the average features of each top level EC class in a normalised descriptor space, and then assigning each instance to the closest class, as measured by the angle between vectors. Their overall headline accuracy figure was only 33.1%. This is stated to be significantly better than random assignment, which they take as the die-rolling value of one sixth, though an accuracy of 28.4% could be obtained by assigning everything to the largest of their unbalanced classes. Dobson & Doig [17] had earlier achieved a similar accuracy of 35% using a SVM model based on sequence and structural features. These figures demonstrate the difficulty of constructing a truly global model for predicting EC from protein structure.

A well-designed answer to the challenge of prediction from sequence has been provided by De Ferrari *et al. *[18] who use a kNN model (with k = 1) along with a very large training set. This means that the information used to make the prediction is extremely local, the single nearest neighbour in a descriptor space built on binary fingerprints of presence and absence of specific InterPro sequence signatures, but the coverage of their model is global. Headline prediction accuracy above 97% was achieved, outperforming other bioinformatics based predictions. This excellent result shows that EC number annotation is highly conserved between neighbouring sequences in their bioinformatics descriptor space and that almost all test sequences had a suitably close homologue available in their large dataset. Their method's predictive success might broadly be ascribed to evolutionary conservation of both protein sequence signatures and catalysed chemical transformation, [26] though Almonacid & Babbitt have demonstrated that over a third of evolutionarily related families of enzymes carry out more than one catalytic function [27]. However, the construction of De Ferrari *et al*,'s method relies on sequence-similar proteins sharing the same functional label, which is analogous to the principles by which such functional annotations are assigned in the first place. We note that any propagation of functional mis-annotation in bioinformatics databases [28] is unlikely to be detected by a neighbour based method. This would register a correct prediction since the training and test instance have the same label; even if that label was actually mis-assigned at some earlier stage in its propagation through the databases.

Now we come to the cheminformatics version of the problem, finding the EC number for a known chemical reaction. Since EC numbers, at least at the first three levels, are ontologically related to the chemical bond making and breaking inherent in the reaction, one might argue that the cheminformatics problem is more about assignment than prediction. That problem, though more simply stated than executed, is to encode an algorithm that mimics the role of the humans charged with deciding upon the EC number for a new reaction. Two papers from the Gasteiger group [22,29] looked at the classification of enzyme transformations using Kohonen maps, SVM, and hierarchical clustering. They worked with physicochemical descriptors relating to the reacting bonds and classified only within classes EC 1 and EC 3. The main accuracy figures reported, between 93.3% and 97.7%, do indeed seem impressive. Latino & Aires-de-Sousa similarly use physicochemical and topological descriptors encoding the bonding changes during a chemical reaction [23]. Their RF predictor, run on a genome-wide set of reactions, gave respective accuracies of 95%, 90% and 85% at the first three levels of the EC classification. Their training set included an example of every available full EC number, analogously to reference [18]. Their work also showed that the predictions became much more reliable if a full balanced description of the reaction was used. Yamanishi *et al. *[21] used a graph theory description of reactants, products and reactants to predict possible EC numbers, also making use of their RDM approach.

The work of Egelhofer *et al. *[25] has close links to the IUBMB nomenclature committee through Dietmar Schomburg, and uses a fairly typical cheminformatics encoding of chemical structure in terms of atom and bond types, with a Tanimoto similarity metric. They found a concordance of over 80% for their method as compared with the EC, over a dataset of 3788 enzymatic reactions (3115 were unambiguously right, 61 existing assignments appear to be technically incorrect in the sense that they break the rules in some way, and the remaining 612 were subject to some other complexity or ambiguity). This illustrates the difficulty of designing an effective automated and algorithmic process to reproduce a classification, EC number, which is occasionally idiosyncratic and inconsistent and whose definition has involved some arbitrary human decision making. Thus, the necessary use of the actual EC classification as a gold standard is a limitation inherent in the validation of these methods. Also in the Schomburg group, Leber *et al. *investigated the correspondence between the EC classification and Dugundji-Ugi R-matrices describing the formal electron changes associated with the reaction [24].

## Methods

### Enzyme reactions and mechanisms

Version 2.4 of the MACiE database of enzyme reaction mechanisms, used in the first part of this study, includes 260 entries. MACiE is unique amongst enzyme databases in combining detailed stepwise mechanistic information with wide coverage of both chemical space and the protein structure universe. MACiE usefully complements both the mechanistic detail of the Structure-Function Linkage Database (SFLD), [30,31] which provides great detail for a small number of enzyme superfamilies, and the wider coverage with less chemical detail provided by EzCatDB [32,33]. We have previously employed MACiE in a major survey of the chemistry involved in enzyme catalysis, [34] to describe quantitatively the similarity between different enzyme reaction mechanisms, [14] to study the geometry of interactions between catalytic residues and their substrates, [35] and to investigate the functional roles of the different residues in enzyme catalysis [36]. MACiE has been extended by incorporating metal ions, [37,38] and by using CMLReact [39] and animations [40]. Newer applications of MACiE are graph theory for identifying atom-atom correspondences between the structures of starting materials, intermediates and products, [41] and the evolution of pathways for cofactor biosynthesis [42].

As we are interested in reaction mechanisms, our dataset is limited to the 260 entries in MACiE 2.4 and the further 60 added in version 3.0, whereas overall reaction based EC number assignment studies have the full set of around 4000 EC numbered reactions to work with. Sequenced-based protein function prediction has hundreds of thousands of annotated sequences available [18].

### Machine learning algorithms

Machine learning is the development of algorithms that enable computers to learn and evolve the behaviours that allow them to interpret data. The many different available machine learning methods are categorised into two main groups: supervised learning where classification is performed based on prior information and unsupervised learning where no information is given prior to learning. We focus on the analysis and comparison of performance of three commonly used supervised approaches, SVM [43], RF [44] and kNN [45] with enzyme mechanistic descriptors derived from the MACiE [10-13] database.

**Figure 1:**
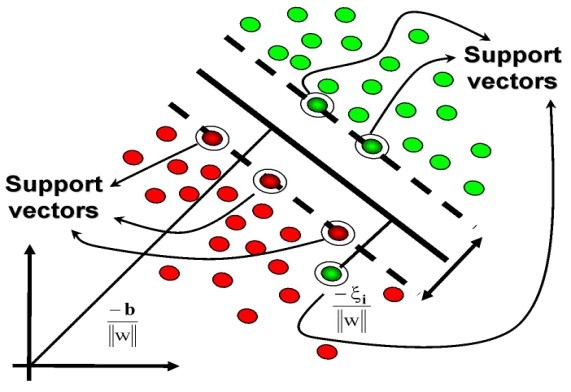
**Linear separating hyperplane for the binary classification**. The solid line shows the maximum margin hyperplane separating the red and green classes. The dotted lines show the margins and the highlighted points are the support vectors.

SVM is a learning method based on statistical learning theory. The SVM maps the inputs into a high-dimensional feature space through a chosen kernel function. The dimensionality of the feature space is determined by the number of support vectors extracted from the training data. In principle, we seek the optimal separating hyperplane between the two classes by maximizing the margin between the closest points (support vectors) (Figure 1). Noble has explained the essence of SVM with respect to four basic concepts [46]: (1) The separating hyperplane, (2) The maximum-margin hyperplane, (3) The soft margin and (4) The kernel function. SVM is used to solve the task of assigning objects to classes.

Linear SVM can be used where two classes are linearly separable. For more complex nonlinear classification problems, the kernel trick [47] (originally proposed by Aizerman *et al*.) provides an elegant and effective way of dealing with this, mapping the data through some nonlinear transformation into a higher dimensional feature space before finding a separating hyperplane in that transformed space with the maximum margin. Common kernel functions include the firstly the polynomial, and secondly the Gaussian radial basis function (RBF).

The various kernel functions contain characteristic hyperparameters that need to be optimised in order to find the best SVM model. In the Gaussian RBF kernel, the number of free hyperparameters to be determined is two: a regularization parameter C which controls the complexity of the decision boundary, and the kernel width σ. Three hyperparameters need to be settled for the polynomial kernel: the regularization parameter C, scale and degree. The advantage of kernel based methods is that once the valid kernel is selected, one can practically work in any dimensionality without paying attention to computational cost. SVM has many applications and it is extensively used for classification problems in bioinformatics and cheminformatics.

A Random Forest is an ensemble of diverse decision trees, generated by sampling the same training data. Trees are constructed by the Classification And Regression Trees (CART) algorithm. Each tree is based on a different randomly selected bootstrap sample of the data; a sample of *N *items from the pool of *N*, chosen *with replacement*. Thus, some items will occur more than once in the sample from which a particular tree is grown. Other items will therefore not be chosen for this bootstrap sample; these form the *out of bag *data for this tree. The probability of omission from a bootstrap sample is (1 - 1/*N*)*^N^*, which tends towards a limit of e^-1 ^≈ 0.37 as *N *becomes large. The second tree is based on a fresh randomly chosen bootstrap sample, and so on throughout the forest.

As a tree is grown, the possible branch splittings are investigated, based on the values of a subset of descriptors. A fresh subset of descriptors is randomly selected at each new node to generate potential splits; we treat the subset size *mtry *as a hyperparameter of the method, but as a default the randomForest package in R uses √*M*, where *M *is the number of descriptors. The best available split (typically chosen according to the Gini impurity criterion) at the given node is used, and branches grown to two child nodes. The tree continues to be grown in this way until no further subdivision is possible; unlike some other decision tree methods, in RF the trees are not pruned.

Trees grown in this way can then be used to predict data they have not seen before. The usual internal validation of a RF is based on the *out of bag *data; each tree is asked to predict the data omitted from its own bootstrap sample. Each item is thus predicted by roughly 37% of the trees in the forest. In RF classification, as relevant to this work, each tree's predicted class assignment is counted as one vote and the forest's consensus prediction for an item is simply that with the most votes; ties are broken at random. Unseen test data are predicted just as for *out of bag *samples, except that every tree now predicts every test item.

Advantages of RF include not having to split the data into separate training and test sets (if *out of bag *validation is used), and especially RF's tolerance of unimportant descriptors. This means that it is not usually necessary to carry out descriptor selection with RF.

The *k*-Nearest Neighbour (kNN) classifier is an instance-based learning algorithm based on the minimum distance(s) from the query instance to the training samples. Descriptors, which if numerical are usually scaled, are used to locate each instance in a multidimensional coordinate space. Euclidean distance (or some other proximity measure) is used to determine the *k*-nearest neighbours in this space, where *k *is a small integer. The class of the query item is then assigned by majority vote amongst the *k *neighbours. In the case of *k *= 1, the class label is therefore copied directly from the nearest neighbour. Instances with the same class label (here, EC class) are expected typically to have smaller separating distances compared to instances belonging to different classes. The kNN algorithm is, perhaps, the simplest among all machine learning methods. Only one parameter in kNN needs to be optimised; this is k, the number of nearest neighbours used to vote for the classification. kNN is a local method, in that the prediction for a test item is determined only by training items close to it, but its coverage is as broad as the diversity of the training set. Even where the descriptors are actually similarity measures, our kNN procedure locates neighbours in the high dimensional descriptor space, rather than just finding the highest similarity value in a row or column.

For parameter tuning we have used the CARET package in the statistical software suite R [48]. The *Train *method creates a grid to evaluate the parameter settings with a fixed step-size through a wide range of values and to assess the performance of every combination. The model with the highest accuracy is selected as the candidate model.

### Five sets of descriptors

Our study uses five sets of numerical descriptors. Three of them encode information derived from the overall chemical transformation. The other two represent information relating to the chemical mechanism by which the transformation occurs.

The *human designed *descriptors belong to the first category since we based them on features of the overall reaction. Many are calculated directly from the overall bond change descriptors; examples are f:X-H, the total number of bonds to hydrogen formed, and dv:C, the total change in the sum of all bond orders to carbon. Other human designed descriptors are derived from the chemical structures of the molecules involved or the equation of the reaction. Some are designed very deliberately to correlate with certain EC classes. For example, water.OH-.su is set to 1 whenever water (or OH^-^) is a substrate; it should take the value 1 for every EC 3.-.-.- reaction, though the converse is not true. The descriptor Mod_Diff is the difference in molecular weight between the largest substrate and largest product, and was designed to be 0.000 for every EC 5.-.-.- reaction (actually, a single counter-example was found due to a protonation state difference). These descriptors are expected to predict EC class better than any of the less artificial types of descriptor. In addition, their development using the training set data means that their performance on internal validation measures, including the RF out-of-bag error, may overestimate their true discriminatory power; the external validation is, however, a fair test of them. Full details of the *human designed *descriptors, and their values, are available in Additional files 1 and Additional file 2.

The *overall bond change *descriptors of Holliday *et al. *[12] list the numbers of covalent bonds between a given pair of elements that are formed, cleaved or changed in order on going from the starting materials to the products. For instance, the descriptor C.N_0.1 is the number of carbon-nitrogen single bonds formed in the reaction, and O.O_2.1 is the number of oxygen-oxygen double bonds in the starting materials that become single bonds in the products. Thus, these descriptors consist of integers describing the number of occurrences of each bond change in each overall transformation. Their values are given in Additional file 3.

The *overall reaction similarity *descriptors of Almonacid *et al. *[15] give the similarity of each overall reaction to each of the 260 entries in MACiE 2.4; for the external validation a further 13 columns give the similarity to the additional training set reactions. The similarity is based on combining bond changes to give Tanimoto scores. The *overall reaction similarity *is based on the similarity of the overall chemical transformation, with no reference to its stepwise mechanism. The *overall reaction similarity *is calculated for both reactions in the canonical direction indicated in MACiE only. In all cross-validation tests, columns of the descriptor matrix corresponding to any entry in the (internal) test set for that cross-validation fold are deleted before training takes place. Note that since RF out-of-bag performance is defined on the training set, the out-of-bag results do not reflect such deletions, though the RF cross-validated results do. Thus, these descriptors consist of real numbers between 0 and 1 expressing the similarity between each pair of overall transformations; see Additional file 1. Their values are given in Additional file 4. While they convey closely related underlying information to the *overall bond change *descriptors, both sets are included here in order to make a comparison between them.

The *composite bond change *descriptors [12] consist of bond change information, analogous to the *overall bond change *descriptors. For the *composite bond change *descriptors, however, these changes are considered for each step, rather than for the overall reaction. The composite descriptors are derived by summing the descriptors corresponding to each step of the reaction. The *composite bond change *descriptors are therefore mechanistic features consisting of integers expressing the number of occurrences of each bond change summed over each step of each reaction. This means that, for example, a C-O single bond formed in one step and broken in a subsequent one will appear as both C.O_0.1 and C.O_1.0 in the *composite bond change *description of the mechanism; see Additional file 1. In contrast, such a transient bond will not appear in the representation of the chemical transformation from substrates to products, and hence would be absent from the *overall bond change *description of the reaction. The values of the *composite bond change *descriptors are given in Additional file 5.

The *mechanistic similarity *descriptors [15] give a different representation of mechanistic information. They give the similarity of each stepwise mechanism to each of the other entries in MACiE. The similarity is obtained by aligning the steps of the two mechanisms using a Needleman-Wunsch alignment procedure, as explained in Almonacid *et al*., [15] and is analogous to sequence similarities obtained *via *sequence alignments. The *mechanistic similarity *is calculated for both reactions in the canonical direction in MACiE, and then recalculated with one reaction reversed; the higher similarity is selected. Thus, these descriptors consist of real numbers between 0 and 1 expressing the similarity between each pair of chemical mechanisms. Their values are given in Additional file 6. Though they express related information to the *composite bond change *descriptors, the two sets are included here in order to facilitate comparison. During cross-validation, column deletion is carried out for *mechanistic similarity*, as described for *overall reaction similarity *above.

A number of individual numerical descriptors are distributed over different ranges, affecting three of the five given descriptor sets. The most strongly affected are the *human designed *descriptors, which included both small integer counts of bond changes and large numbers corresponding to molecular weights. We anticipated that kNN would be particularly vulnerable to differences in descriptor ranges, with the larger valued descriptors dominating computations of distance. Hence, we scaled all descriptors in the *overall bond change, human designed*, and *composite bond change *sets such that each had a mean of 0.0 and a standard deviation of 1.0. The *overall reaction similarity *and *mechanistic similarity *descriptors are by definition all real numbers between 0 and 1, and are not further scaled.

### Cross-validation strategy

**Figure 2:**
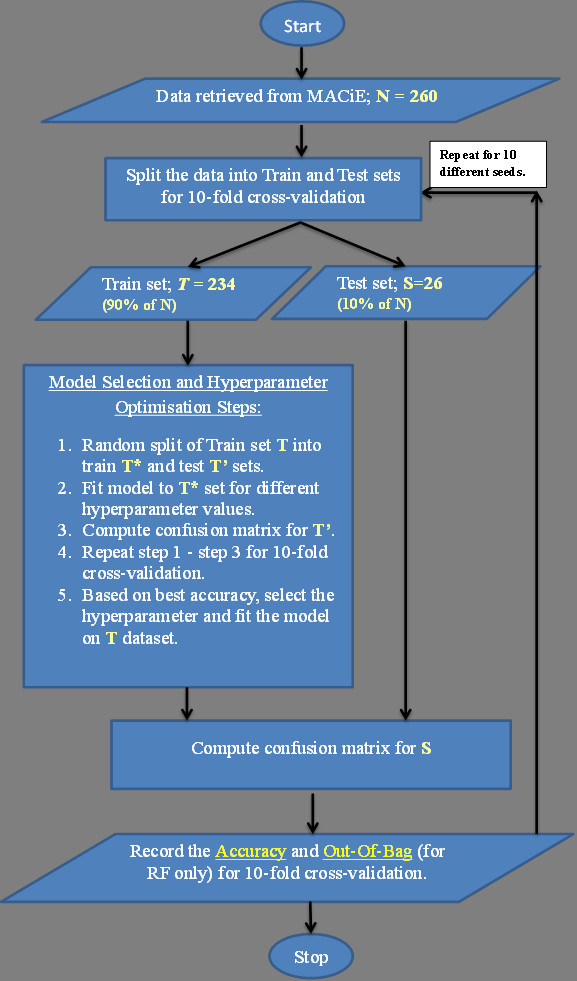
**Workflow of the cross-validation exercise**. Flow chart illustrating the workflow used in the cross-validation part of this study.

There are many methods for estimating the performance of machine learning such as the hold-out method, leave-one-out, and k-fold cross-validation. In the first part of this study, we performed 10 fold cross-validation to estimate classifier performance. The cross-validation involves training and prediction procedures in which the class instances were randomly distributed into 10 folds, where nine out of 10 were used as a training set, and the remaining one as the test set. N-fold cross-validation has been widely accepted as a reliable method for calculating generalization accuracy and experiments have shown that cross-validation is relatively unbiased [49]. Our procedure is illustrated in the flow chart of Figure 2. In addition to the cross-validation results, we also calculate the out-of-bag accuracy for the RF models.

Accuracy, calculated as the proportion of the predictions made which are correct, is an easily understood and very widely used measure of predictive power. Nonetheless, it may not be a good measure for data which are unbalanced between classes, and a prediction based simply on assigning instances to the largest class might score a reasonable accuracy without being of any practical use. In our work, the *train *procedure selects models based on accuracy in an internal validation, and hence our predictions may show a small bias towards larger classes. Mindful of this, we also present values of Gorodkin's K-category correlation coefficient R_K_, [50] which is an extension of the Matthews correlation coefficient [51] to multi-class data and gives a more balanced measure of prediction success across classes.

### External validation set

During the course of this study, MACiE version 3.0 was released, containing a further 60 entries. This presented us with the opportunity of generating an external test set in addition to the existing cross-validation. To ensure that the test set was reasonably representative, it was chosen by construction to be non-redundant at the third level of the EC classification. This led to a test set of 43 entries, while the 17 third level EC duplicates amongst the 60 new MACiE entries were added to the training set for this validation exercise; the training set thus comprised 277 entries. Training set descriptors for the *human designed, overall bond change *and *composite bond change *sets were scaled as described above, and the equivalent test set descriptors were scaled using the corresponding training set distributions.

### Significance tests

We assessed the statistical significance of the difference in prediction performance between each pair of methods for a given descriptor set, and between each pair of descriptor sets for a given method. For a given pair of classifiers and definition of cross-validation folds, the difference is given by

$$D_{0}=\left(N_{A}^{1}-N_{B}^{1}\right)+\left(N_{A}^{2}-N_{B}^{2}\right)+\left(N_{A}^{3}-N_{B}^{3}\right)...+\left(N_{A}^{10}-N_{B}^{10}\right),$$

where $N_{A}^{1}$ is the number of correct predictions made by classifier *A *for fold *1 *and so on. We used a permutation test as described by Menke & Martinez, [52] in which 1024 permutations were created via all 2^10 ^combinations of

$$D_{p}=\pm \left(N_{A}^{1}-N_{B}^{1}\right)\pm \left(N_{A}^{2}-N_{B}^{2}\right)\pm \left(N_{A}^{3}-N_{B}^{3}\right)...\pm \left(N_{A}^{10}-N_{B}^{10}\right).$$

The rank of the true difference in performance (*D_0_*, the difference when all signs are +) amongst the population of values obtained by the 1024 permutations is used as an indicator of the p-value; the probability that such an extreme value would be obtained if there were no intrinsic difference between the classification abilities of the two classifiers. This equates to

$$p=\frac{n}{1024},$$

where *n *is the number of permutations which give

$$\left|D_{p}\right|\geq \left|D_{0}\right|.$$

The value of *n *can never be smaller than 2, since both *D_0 _* and -*D_0 _* must be found amongst the set of differences {*D_p_*}.

## Results

The cross-validation results are given in Tables 1 (accuracy) and 2 (R_K_). We find that the three descriptor sets encoding overall chemical transformation perform better than the two descriptions of mechanism. Thus, amongst directly comparable pairs of descriptors, *overall reaction similarity *always gives better predictive accuracy than *mechanistic similarity*. Similarly, *overall bond change *descriptors give better accuracy than *composite bond change*. Thus, mechanism cannot be used as a proxy for chemical reaction in assigning an EC number. This seems to reinforce the conclusion of Almonacid *et al. *[15] that pairs of similar (but non-homologous) enzyme reactions tend to proceed by different mechanisms; hence EC class is more accurately predictable from similarity of chemical transformation than from similarity of mechanism.

**Table 1:** Cross-validation accuracy

Package	CARET, Train			randomForest	
**Method**	**SVM (RBF)**	**SVM (poly)**	**kNN**	**RF**	**RF-OOB**

*Human Designed*	0.865	0.883	0.787	0.907	0.910

*Overall Bond Change*	0.681	0.640	0.618	0.682	0.682

*Overall Reaction Similarity*	0.714	0.703	0.666	0.708	0.707

*Composite Bond Change*	0.623	0.611	0.557	0.614	0.616

*Mechanistic Similarity*	0.598	0.574	0.515	0.567	0.566

Average cross-validated accuracy over 10 repetitions of 10-fold cross-validation, as shown in Figure 2, for four methods and five descriptor sets

The *human designed *descriptors were specifically constructed to facilitate the identification of the correct EC class from the other information available in a MACiE entry. These 28 descriptors seek to recognise characteristic features of reactions in each EC class, such as: involvement of known oxidising and reducing species (EC class 1), water (EC class 3), starting material and product being isomers (EC class 5), and hydrolysis of ATP (EC class 6). These allow a classification accuracy of 91% with RF; more exhaustive development of these descriptors would lead us closer to an algorithmic cheminformatics implementation of the definition of EC class [23-25].

The second and third best performing sets, *overall reaction similarity *and *overall bond change *descriptors, both carry information about the overall chemical transformation. While it is unsurprising that neither can match the *human designed *set's results, all three encodings of overall chemical transformation outperform both descriptions of mechanism, of which the *composite bond change *descriptors are slightly more effective than the more sophisticated alignment-based *mechanistic similarity *descriptors of Almonacid *et al*.

For all four machine learning classifiers, the ranking of the five descriptor sets is identical, and in every case the differences between all pairs of descriptors are statistically significant:

Human Designed > Overall Reaction Similarity > Overall Bond Change > Composite Bond Change > Mechanistic Similarity.

**Figure 3:**
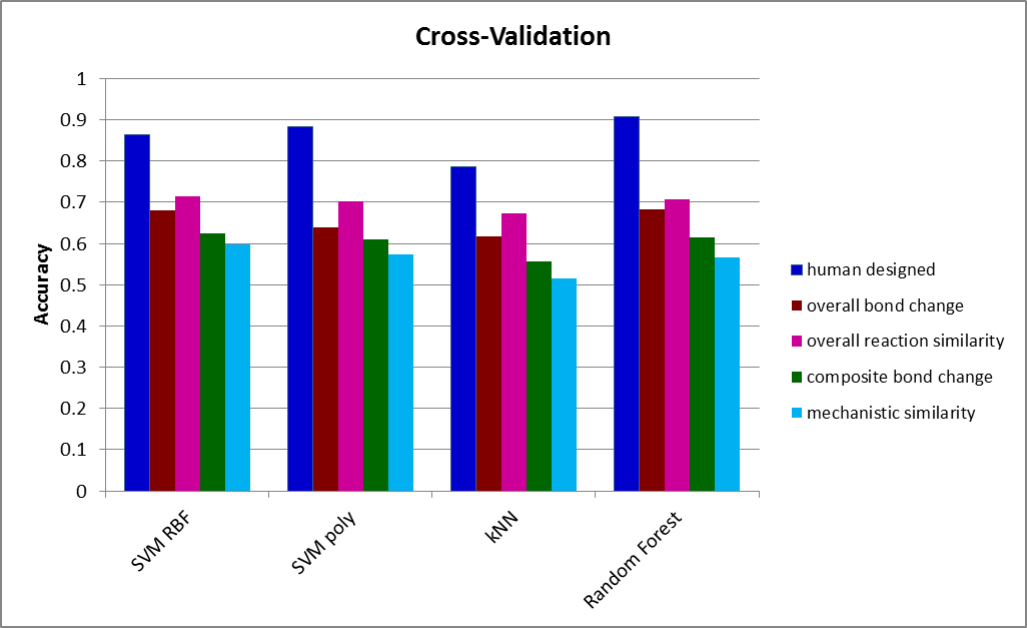
**Performance of different classifiers in cross-validation**. The Figure shows the accuracy achieved by each of the four classifiers for each of the five descriptor sets in the cross-validation.

Tables 1 and 2 also show that the two different SVM kernels and RF all produce similar quality predictions, though the kNN predictions are generally less successful. The relative performance of the methods varies somewhat with the descriptor set used, and is illustrated in Figure 3. Taking into account the statistical significance tests, the relative performance (where ≈ indicates no statistically significant difference at the 5% level) was as follows.

**Table 2:** Cross-validated values of Gorodkin's R_K_

Package	CARET, Train			randomForest
**Method**	**SVM (RBF)**	**SVM (poly)**	**kNN**	**RF**

*Human Designed*	0.831	0.853	0.737	0.884

*Overall Bond Change*	0.596	0.547	0.525	0.600

*Overall Reaction Similarity*	0.639	0.625	0.579	0.631

*Composite Bond Change*	0.522	0.509	0.443	0.510

*Mechanistic Similarity*	0.489	0.457	0.379	0.447

Average cross-validated value of Gorodkin's K-category correlation coefficient over 10 repetitions of 10-fold cross-validation, as shown in Figure 2, for four methods and five descriptor sets

*Overall bond change; *RF ≈ SVM (RBF) > SVM (poly) > kNN.

*Human designed; *RF > SVM (poly) > SVM (RBF) > kNN.

*Overall reaction similarity; *SVM (RBF) ≈ RF ≈ SVM (poly) > kNN; SVM (RBF) > SVM (poly).

*Mechanistic similarity; *SVM (RBF) > SVM (poly) ≈ RF > kNN.

*Composite bond change; *SVM (RBF) ≈ RF ≈ SVM (poly) > kNN.

Table 3 shows a class-by-class analysis of the prediction accuracy. This shows firstly that the classes differ substantially in their prediction difficulty, and secondly that the ordering of descriptor sets by accuracy changes between classes. This is discussed in more detail below.

**Table 3:** Prediction accuracies by EC class

Package & Method	randomForest, Random Forest					
**EC Class**	**1**	**2**	**3**	**4**	**5**	**6**

*Human Designed*	0.961	0.816	0.948	0.835	0.952	0.877

*Overall Bond Change*	0.823	0.394	0.849	0.605	0.500	0.731

*Overall Reaction Similarity*	0.865	0.500	0.828	0.568	0.628	0.600

*Composite Bond Change*	0.870	0.406	0.680	0.495	0.276	0.654

*Mechanistic Similarity*	0.817	0.363	0.722	0.334	0.333	0.315

Prediction accuracies by EC class and descriptor type. These data are for the RF method as implemented in the R package randomForest [48].

**Figure 4:**
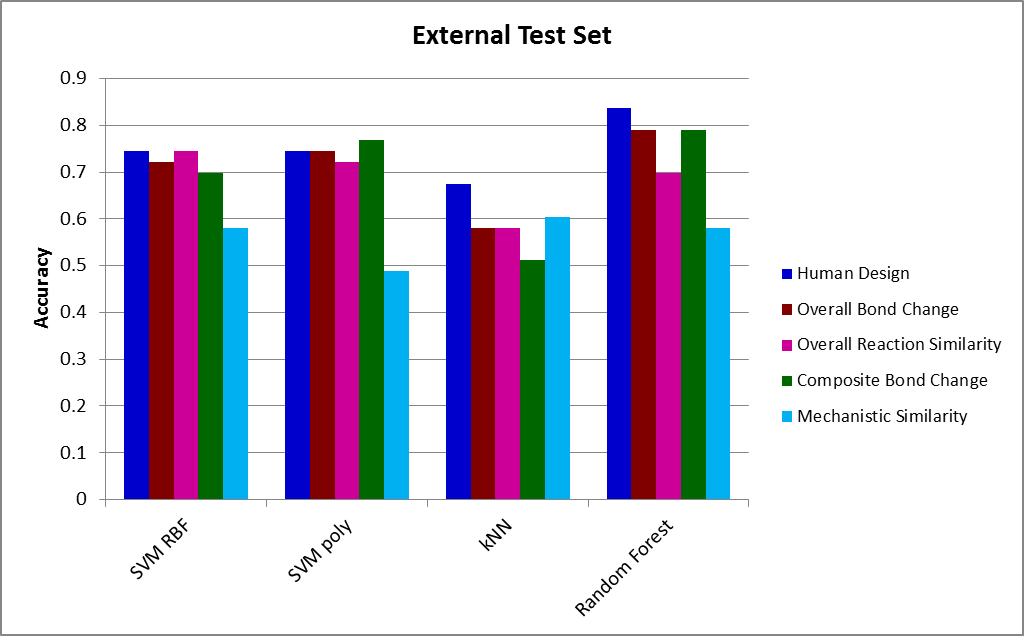
**Performance of different classifiers for the external test set**. The Figure shows the accuracy achieved by each of the four classifiers for each of the five descriptor sets for the external test set.

Table 4 shows the results for MACiE version 3, split into a 277-entry training set (including all the 260 entries from version 2 plus 17 third level EC number duplicates from amongst the new entries) and a non-redundant 43-entry test set. The performance of the classifiers on the external test set is illustrated in Figure 4.

**Table 4:** External test set accuracy

Package	CARET, Train			randomForest
**Method**	**SVM (RBF)**	**SVM (poly)**	**kNN**	**RF**

*Human Designed*	0.744	0.744	0.674	0.837

*Overall Bond Change*	0.721	0.744	0.581	0.791

*Overall Reaction Similarity*	0.744	0.721	0.581	0.698

*Composite Bond Change*	0.698	0.767	0.512	0.791

*Mechanistic Similarity*	0.581	0.488	0.605	0.581

Prediction accuracy on the 43-entry external test set for four methods and five descriptor sets.

## Discussion

Almonacid *et al. *showed that, for convergently evolved pairs of enzymes sharing an EC sub-subclass, *overall reaction similarity *was almost universally higher than *mechanistic similarity *[15]. In our current work, *overall bond change *descriptors can predict EC class with up to 68% accuracy, compared to 62% for the mechanism dependent *composite bond change *description of the reaction. *Overall reaction similarity *gives 71% prediction accuracy, *mechanistic similarity *only up to 60%. Thus, we find that the descriptor definitions based on overall reaction tend to be better predictors than those based on chemical mechanism, though *Composite Bond Change *does well on the external test set. Since EC numbers are defined on the basis of the overall chemical transformation catalysed, the strong performance of overall reaction-based measures is reassuring - albeit that some questions arise over the congruence of the EC sub-subclass-based and descriptor-based definitions of a "similar reaction". In the present work, we are necessarily looking at predicting the top level EC class, since MACiE contains insufficiently many examples of each category at the subclass or lower levels. Hence the overall reactions sharing the same label in this study are considerably less similar than those sharing third or fourth level labels. Nonetheless, the superiority of the predictions made using descriptors based on overall chemical transformation is entirely in accordance with the conclusions from [15]; mechanisms of analogous reactions tend to be less similar than are the overall reactions.

EC class 1, the oxidoreductases, covers a diversity of chemistry - the unifying feature being that all are redox reactions. Within the breadth of biological redox reactions, there are some recognisable clusters: significant proportions of these reactions involve interconversion of NAD and NADH, or NADP and NADPH (ten and 14, respectively, out of the 84 oxidoreductases in MACiE 3.0). Overall, the oxidation and reduction reactions of EC class 1 seem to leave recognisable chemical signatures in the descriptors; for instance C-H cleavage, O-H formation and C-C bond order changes are all common, and class 1 is generally well-recognised. Our chemical interpretations here, and indeed for all six classes, are based on analysis of both descriptor values and RF variable importance scores.

EC class 2, transferases, encompasses any chemical reaction that transfers a functional group from one molecule to another; quite commonly phosphate moieties, in the cases of kinases and phosphatases, or methyl groups are transferred. The 63 MACiE 3.0 entries in EC class 2 are diverse reactions, seeming to lack clear chemical patterns. Unsurprisingly, they are poorly predicted.

EC class 3, the hydrolases, is more tightly defined than many of the other classes, since it consists of reactions where water is used to hydrolyse a chemical bond. In fact, two of our 65 hydrolases are exceptions to this rule: M0226 is annotated as the reverse reaction, while M0172 is presented as utilising a hydroxide ion rather than neutral water. Almost half of the hydrolases in MACiE 3.0, 33 out of 73, catalyse the hydrolysis of biopolymers such as peptides, proteins, DNA or RNA. Hydrolases are well-predicted by all the descriptor sets, though less so for *composite bond change *descriptors. Hydrolysis leads to simple repeated and recognisable patterns of bond making and breaking. An example for the *overall bond change *is C-N single bond cleavage, combined with C-O and N-H single bond formation, for amide or peptide hydrolysis. These are recognisable from both overall reaction and, to a slightly lesser extent, mechanistic data. In the mechanistic case, the corresponding patterns also include bond changes which occur in one step of the mechanism and are subsequently undone in a later step.

EC class 4, lyases, includes those enzymes that catalyse the breaking of a covalent bond, other than by redox or hydrolysis reactions. While a typical text book definition of a lyase may specify that there should be one substrate and two products, only 28 of the 49 lyases in MACiE 3.0 obey this rule. Six are presented as the reverse reaction, and as many as 15 present an assortment of stoichiometric or other complexities. Despite these extra challenges, lyases are generally well-recognised as overall reactions, primarily due to the prevalence of C-C single bond cleavage and C-H single bond formation.

EC class 5, isomerases, comprises enzymes that catalyse a reaction in which the product is an isomer of the starting material. Twenty seven of the 30 examples in MACiE 3.0 have the simple stoichiometry of one starting material being transformed into one isomeric product; 19 of the 30 enzymes catalyse constitutional isomerisation, seven are epimerases or racemases, two topoisomerases catalyse winding or unwinding of DNA, one enzyme is a cis-trans isomerase and one a tautomerase. We find that isomerases are well predicted by overall reaction descriptors, but are extremely hard for the mechanistic descriptors to predict. We interpret the lack of a relationship between membership of EC class 5 and mechanism as indicating that the class comprises a diversity of reactions, united only by the feature that the product is an isomer of the starting material. Thus, our results support the hypothesis that isomerisation reactions can evolve from mechanistically diverse starting points. The two overall reaction based descriptors do rather better, possibly because the reactions often involve formation or cleavage of O-H single bonds. Given knowledge of the definition of an isomerase as an enzyme whose substrate and product are isomers, it is a simple matter for a human to design a cheminformatics descriptor or descriptors to capture isomerisation reactions. The *human designed *descriptors were deliberately engineered to include the change in molecular mass between the largest substrate and the largest product. This descriptor is zero for all but one of the isomerases and allows this descriptor set to recognise isomerases with high accuracy. The isomerase most often incorrectly predicted by the *human designed *predictions is M0196, where the starting material and product are a (trivial) protonation state away from being isomers.

EC class 6, ligases, is composed of enzymes that catalyse the joining together of two molecules coupled with the conversion of ATP to AMP, or ATP to ADP. The *human designed *descriptors are chosen so that they specifically include a feature recognising ATP hydrolysis; this allows them to recognise ligases accurately. Ligases are characterised by both the formation and cleavage of P-O single bonds and we suggest this as the reason why both the *overall *and *composite bond change *descriptors do well in recognising ligases.

As expected, the *human designed *descriptors fared less well on the external test set than in the cross-validation. Unlike the other descriptor sets, the *human designed *descriptors were developed and tested specifically to predict EC class at a time when version 2.4, but not version 3.0, of MACiE was available. This illustrates what we believe to be a general principle, that prediction and classification methods tend to do better on data that were available while they were being developed, and less well on prospective or blind tests. We note also that the class balance of the 43-reaction test set differed from that of the previous cross-validation set. The other descriptor sets, which were defined by their original authors and not developed for classification in the light of available data, generally perform somewhat better on the external test. The *overall bond change *and *composite bond change *methods do relatively well on the external test set.

It is common in the literature to compare the performance of different machine learning methods. We believe that the relative performance depends on context. Some studies have reported that SVM outperforms RF, [53,54] while others suggest that RF and SVM give very similar prediction quality [55,56]. Our results here suggest that there is no significant difference: both SVM (RBF) and RF achieve an average accuracy of 0.696 across the whole cross-validation exercise. kNN is clearly less effective in this work, but extremely good in a different EC prediction context [18]. kNN is an essentially local method with global coverage and, where class labels are strongly conserved in small regions of descriptor space, will prove a good methodology. For the current problem, the strategy of non-redundancy used in populating MACiE means that most MACiE entries do not have a very close neighbour and kNN is less useful.

## Conclusions

Our results strongly suggest that different enzymes typically bring about similar chemical transformations by dissimilar mechanisms. We conclude this because we find that the use of mechanistic information in a set of descriptors encoding an enzyme reaction diminishes its EC prediction performance relative to analogous descriptors with information only on the overall transformation. Thus, *composite bond change *[12] descriptors do significantly less well than *overall bond change; *similarly, *mechanistic similarity *[15] is outperformed by *overall reaction similarity*.

We observe that oxidoreductases and hydrolases have distinctive signatures which are relatively easy to recognise in cheminformatics descriptors; these classes can be identified accurately from overall reaction descriptors and fairly accurately from mechanism. If we specifically design descriptors to recognise cases where the product is an isomer of the substrate, we can identify isomerases. However, isomerases are very hard to assign from mechanism alone; we find no common mechanistic patterns amongst EC class 5. Rather, a diversity of mechanistically quite different enzymes comprise the isomerases, catalysing a disparate group of reactions united only by one property: the substrate and product being isomers. A specific feature to identify ATP hydrolysis allows our *human designed *descriptors to identify ligases. Transferases and lyases are hardest to recognise, without any clear chemical signature in either the overall reaction or mechanistic data.

The performance of the various machine learning algorithms is in line with many cheminformatics applications, with SVM and RF performing about equally well in the cross-validation. RF does slightly better than SVM on the external test set. The relatively modest performance of kNN illustrates the importance of non-local information for recognising EC class from reaction, as opposed to protein, information.

We note also that, despite a lack of clarity in the literature, EC number prediction is not a single problem. The challenge of predicting protein function from available sequence data is quite different from assigning an EC classification from a cheminformatics representation of a reaction. The wide range of prediction success, with headline figures between 33% and 98% in the literature, tells us as much about the difficulties of prediction from different data sources as about the quality of various machine learning algorithms.

## Competing interests

The authors declare that they have no competing interests.

## Authors' contributions

NN designed the workflow, carried out the majority of the computations, performed the statistical analysis and participated in the drafting of the manuscript. JM conceived the study, carried out some computations and participated in the drafting of the manuscript. Both authors read and approved the final manuscript.

## Supplementary Material

Additional file 1 **Details of the five sets of descriptors used in this work**. Click here for file

Additional file 2 **The values of the *human designed *descriptors used in this work**. Click here for file

Additional file 3 **The values of *overall bond change *descriptors used in this work**. Click here for file

Additional file 4 **The values of *overall reaction similarity *descriptors used in this work**. Click here for file

Additional file 5 **The values of *composite bond change *descriptors used in this work**. Click here for file

Additional file 6 **The values of *mechanistic similarity *descriptors used in this work**. Click here for file
