# Supplementary material for: Is EC class predictable from reaction mechanism?
Source: BMC Bioinformatics. 2012 Apr 24;13:60. doi: 10.1186/1471-2105-13-60 (PMC3368749; doi:10.1186/1471-2105-13-60)
Supplement: Additional file 1 — Details of the five sets of descriptors used in this work. [file 1471-2105-13-60-S1.DOC]

# Is EC class predictable from reaction mechanism?

### Neetika Nath, John B O Mitchell

**Additional File 1:** Notes on the descriptors

*We list the descriptor sets in the order that most easily allows us to explain the relationships between them.*

**Overall Bond Change**

The *overall bond change* descriptors give the numbers of bond changes of each type in each overall chemical transformation. They are explained by the example of these columns detailing bonds between carbon and nitrogen …

**C.N_0.1** is the number of C-N single bonds formed.

**C.N_1.0** is the number of C-N single bonds cleaved.

**C.N_1.1** is the number of C-N single bonds that undergo a purely stereochemical change.

**C.N_1.2** is the number of C-N single bonds in the starting materials that become double bonds in the products.

**C.N_2.1** is the number of C-N double bonds in the starting materials that become single bonds in the products.

The exact number of columns depends on which changes occur at least once in the dataset. The *overall bond change* descriptors depend only on the chemical structures of the starting and finishing points of the process and hence do not depend on mechanism.

**Composite Bond Change**

The *composite bond change* descriptors are conceptually similar to the *overall bond change* ones, but now give the total number of times each bond change occurs, summed over each of the individual steps of the reaction.

This means that if a C-N single bond is formed in step 1, broken in step 2 and reformed in the final step, step 4, these changes are represented as follows

**C.N_0.1** takes the value 2 (compared with 1 in the *overall bond change* descriptors).

**C.N_1.0** takes the value 1 (compared with 0 in the *overall bond change* descriptors).

This means that the *composite bond change* descriptors reflect the mechanism by which the change occurs.

**Overall Reaction Similarity**

The *overall reaction similarity* descriptors consist of a Tanimoto similarity measure in the range 0 to 1 for each pair of MACiE entries. The *overall reaction similarity* is calculated for both reactions in the canonical direction indicated in MACiE only. The similarity uses the *overall bond change* values for each of the two reactions, as described in detail in Figure 1(a) of Almonacid *et al.*, cited as reference [15] in our paper. They were calculated using software kindly provided by Dr Daniel Almonacid. The version of the file supplied here gives the similarity of each overall reaction to each of the 260 entries in MACiE 2.4 and also to the 13 additional training set reactions from MACiE 3.0 used to train the models for the external validation experiment.

**Mechanistic Similarity**

The *mechanistic similarity* descriptors also consist of a similarity measure in the range 0 to 1 for each pair of MACiE entries. Mechanistic similarity requires an initial global alignment of the sets of mechanistic steps comprising each reaction. Firstly, the Tanimoto similarity between each pair of steps is written to a matrix. Secondly, the Needleman-Wunsch algorithm is used to carry out an alignment, analogously to aligning sequences in bioinformatics. Finally, the mechanistic similarity is calculated as a new Tanimoto score based on numbers of steps in the reactions and the alignment score. This process is described in detail in Figure 1(b) of Almonacid *et al.*, cited as reference [15] in our paper. The *mechanistic similarity* is calculated for both reactions in the canonical direction in MACiE, and then recalculated with one reaction reversed; the higher similarity is selected.

**Human Designed**

The *human designed* descriptors were engineered to obtain the maximum predictive ability for EC class for relatively modest effort in inputting data. Many of the *human designed* descriptors can be calculated algorithmically and automatically from *overall bond change* descriptors at almost no cost in terms of human or computational time. Some others require input information from the MACiE entries; for the purposes of this single study, not all of this was automated. The *human designed* descriptors are expected to predict EC class better than any of the less artificial types of descriptor, as they lie conceptually somewhere between prediction and chemoinformatics-based assignment. In addition, their development using the training set data means that their performance on internal validation measures, including the random forest out-of-bag error, may overestimate their true discriminatory power; the external validation is, however, considered to be a fair test of them. Descriptions of the 28 *human designed* descriptors follow.

**Sum** is the total number of bond changes of any kind occurring in the overall reaction. It includes formation, cleavage and order changes. It is calculated algorithmically.

**SignedSum** is the sum of the signed bond changes, such that formation of a single bond contributes +1 and cleavage of a single bond contributes -1. An order change contributes the numerical value of the change in order; thus an order change from single to double contributes +1, a change from triple to single contributes -2 and so on. It is calculated algorithmically.

**f:X-H** is the number of single bonds to hydrogen formed in the reaction. It is calculated algorithmically.

**c:X-H** is the number of single bonds to hydrogen cleaved in the reaction. It is calculated algorithmically.

**f:sing** is the number of single bonds formed in the reaction. It is calculated algorithmically.

**c:sing** is the number of single bonds cleaved in the reaction. It is calculated algorithmically.

**increase** is the number of bonds increasing their bond order by one in the reaction. It is calculated algorithmically.

**decrease** is the number of bonds decreasing their bond order by one in the reaction. In one case, a triple bond reduces in order to a single bond – this change increments decrease by 2. The descriptor is calculated algorithmically.

**invM** indicates when the overall reaction involves change in the oxidation state of a metal. In the overwhelming majority of cases, metal ions are catalysts and start and finish the reaction in the same oxidation state. There are two cases in MACiE 3.0, M0277 and M0297, where mercury changes its oxidation state by two and the descriptor has the value 2. Otherwise, it is always zero. It is calculated algorithmically.

**stereo** indicates the number of bond changes that are recorded in the overall reaction with a change in stereochemistry, but no change in bond order. It is calculated algorithmically.

**dv:C** is the change in the total summed bond order of all bonds to carbon in the overall reaction. It is calculated algorithmically.

**dv:H** is the change in the total summed bond order of all bonds to hydrogen in the overall reaction. It is calculated algorithmically.

**dv:N** is the change in the total summed bond order of all bonds to nitrogen in the overall reaction. It is calculated algorithmically.

**dv:O** is the change in the total summed bond order of all bonds to oxygen in the overall reaction. It is calculated algorithmically.

**dv:P** is the change in the total summed bond order of all bonds to phosphorus in the overall reaction. It is calculated algorithmically.

**dv:S** is the change in the total summed bond order of all bonds to sulphur in the overall reaction. It is calculated algorithmically.

**dv:max** is the maximum of the moduli of the preceding six “change in the total summed bond order” descriptors. It is calculated algorithmically.

**stoi#subs** is the total number of molecules on the left hand side of the overall reaction equation, taking stoichiometric coefficients into account. For example, an overall reaction with the equation **3A + 2B + C → 2D +2E + F** would give a stoi#subs value of 6. It is presently assigned from a manually generated list of all the substrates and products of each MACiE entry.

**stoi#prod** is the total number of molecules on the right hand side of the overall reaction equation, taking stoichiometric coefficients into account. For example, an overall reaction with the equation **3A + 2B + C → 2D +2E + F** would give an stoi#prod value of 5. It is presently assigned from a manually generated list of all the substrates and products of each MACiE entry.

**stoi#diff** is defined as stoi#subs minus stoi#prod, and so is the change in the total number of moieties in the reaction. Once the previous two descriptors are manually assigned, it is then trivial to obtain.

**water.OH-.su** is set to 1 whenever water (or OH^-^) is a substrate on the left hand side of the overall reaction equation; it should take the value 1 for every EC 3.-.-.-reaction, though the converse is not true. Any species with a nearest-integer molecular mass of zero, such as a photon, is ignored. It is presently assigned manually from the MACiE entries.

**water.OH-.pr** is set to 1 whenever water (or OH^-^) is a product on the right hand side of the overall reaction equation. Any species with a nearest-integer molecular mass of zero, such as a photon, is ignored. It is presently assigned manually from the MACiE entries.

**Mod_Diff** is the difference in molecular weight between the largest substrate and largest product; it was designed to be 0.000 for every isomerase, EC class 5, reaction (actually, a single counter-example was found due to a protonation state difference). It is presently assigned from a manually generated list of all the substrates and products of each MACiE entry.

**O2.Fe2.red.ox.su.pr** indicates the presence of molecular oxygen, Fe^2+^, or any moiety whose description includes the words “oxidised” or “reduced” amongst either the substrates or products in the overall reaction equation. Whenever it takes the value 1, the reaction is expected to belong to EC class 1, oxidoreductases. The converse is not true. It is presently assigned manually from the MACiE entries.

**xTP_hyd** indicates that the reaction involves the conversion of ATP to either ADP or AMP, or alternatively an analogous process involving another nucleoside triphosphate. It was designed to take the value 1 for every ligase, EC class 6, reaction; the converse is not true. It is presently assigned manually from the MACiE entries.

**stoi2#su** is identical to stoi#subs, other than that it excludes species containing only hydrogen. It is presently assigned from a manually generated list of all the substrates and products of each MACiE entry.

**stoi2#pr** is identical to stoi#prod, other than that it excludes species containing only hydrogen. It is presently assigned from a manually generated list of all the substrates and products of each MACiE entry.

**stoi2#diff** is defined as stoi2#su minus stoi2#pr, and so is the change in the total number of non-hydrogen moieties in the reaction. Once the previous two descriptors are manually assigned, it is then trivial to obtain.

**stoi2#mod_diff** is the modulus of stoi2#diff. Once stoi2#su and stoi2#pr are manually assigned, it is then trivial to obtain.

**dssizeratio** is computed as Mod_Diff divided by the molecular weight of the largest substrate. Thus an isomerisation reaction would give a value of 0; cleaving a large molecule into two approximately equally sized parts would give a value close to 0.5; joining two approximately equally sized molecules to form one larger product would give a value close to 1. It is presently assigned from a manually generated list of all the substrates and products of each MACiE entry.

Given the different natures of these descriptors, it may be of some interest to list the descriptor importance values. This are ranked according to the loss of out-of-bag accuracy when the descriptor is replaced by random noise for the 260 MACiE 2.4 entries; the figure quoted is the loss of accuracy normalized by the standard error.

| 1. water.OH-.su | 9.36 |
| --- | --- |
| 1. stoi#subs | 7.78 |
| 1. stoi2#su | 7.45 |
| 1. Mod_Diff | 7.35 |
| 1. O2.Fe2.red.ox.su.pr | 6.88 |
| 1. dssizeratio | 6.55 |
| 1. stoi#prod | 6.33 |
| 1. stoi2#pr | 6.26 |
| 1. decrease | 6.11 |
| 1. xTP_hyd | 6.08 |
| 1. stoi2#mod_diff | 5.98 |
| 1. stoi2#diff | 5.72 |
| 1. increase | 5.69 |
| 1. c:single | 4.97 |
| 1. stoi#diff | 4.97 |
| 1. Sum | 4.93 |
| 1. f:X.H | 4.49 |
| 1. c:X.H | 4.48 |
| 1. dv:max | 4.19 |
| 1. f:single | 4.06 |
| 1. dv:H | 3.89 |
| 1. water.OH-.pr | 2.86 |
| 1. SignedSum | 2.76 |
| 1. stereo | 1.69 |
| 1. dv:N | 1.42 |
| 1. dv:S | 1.34 |
| 1. dv:C | 1.17 |
| 1. dv:O | 1.06 |
